# Evaluation of Arsenic and Cobalt Levels in Pediatric Patients Receiving Long-Term Parenteral Nutrition

**DOI:** 10.3390/nu16081179

**Published:** 2024-04-16

**Authors:** Hanna Romanowska, Aleksandra Wilk, Mikołaj Danko, Anna Borkowska, Katarzyna Popińska, Marta Sibilska, Joanna Żydak, Wojciech Marciniak, Agnieszka Szlagatys-Sidorkiewicz, Janusz Książyk

**Affiliations:** 1 Department of Pediatrics, Endocrinology, Diabetology, Metabolic Diseases and Cardiology of the Developmental Age, Pomeranian Medical University, 71-252 Szczecin, Poland; 2 Department of Histology and Embryology, Pomeranian Medical University, 70-111 Szczecin, Poland; aleksandra.wilk@pum.edu.pl; 3 The Children’s Memorial Health Institute, Department of Pediatrics, Nutrition and Metabolic Diseases, 04-730 Warsaw, Poland; m.danko@ipczd.pl (M.D.); kasia.popinska@wp.pl (K.P.); msibilska@poczta.onet.pl (M.S.); janusz@ksiazyk.pl (J.K.); 4 Department of Pediatrics, Gastroenterology, Allergology and Nutrition, Medical University of Gdańsk, 80-803 Gdańsk, Poland; anna.borkowska@gumed.edu.pl (A.B.); agnieszka.szlagatys-sidorkiewicz@gumed.edu.pl (A.S.-S.); 5 Department of Genetics and Pathology, Pomeranian Medical University, 71-252 Szczecin, Poland; wojciech.marciniak@read-gene.com

**Keywords:** arsenic, cobalt, trace elements, parenteral nutrition, children

## Abstract

This study continues the research in which we determined the concentration of aluminum in children receiving long-term parenteral nutrition (LPN). Since our results were interesting, we decided to assay arsenic (As) and cobalt (Co) in the collected material, which, like aluminum, constitute contamination in the mixtures used in parenteral nutrition. Excesses of these trace elements in the human body are highly toxic, and deficiencies, particularly in the case of Co, can lead to various complications. The aim of this study was to determine the impact of LPN in children on their serum levels of As and Co, as well as the excretion of these elements in urine, and to compare them with a control group of healthy children. The study group consisted of 83 children receiving home parenteral nutrition from two Polish centers, while the control group included 121 healthy children. In both groups, the levels of As and Co in serum and urine were measured. The elemental compositions of the samples were determined using inductively coupled plasma mass spectrometry (ICP-MS). It was demonstrated that the children receiving LPN did not have increased As exposure compared to the controls. Greater exposure compared to the control group was shown for Co. In conclusion, children receiving LPN are not exposed to As, and even though the concentrations of Co in serum and urine were higher in the LPN group than in the healthy controls, neither trace element poses a health threat to children requiring LPN.

## 1. Introduction

Parenteral nutrition (PN) is often a life-saving therapy in patients with intestinal failure. The most common reason for implementing long-term parenteral nutrition (LPN) is short bowel syndrome [1].

In addition to macronutrients (carbohydrates, proteins, and lipids) serving as energy sources, patients receiving PN need an adequate supply of trace elements (TEs) and vitamins, which are essential for the body to function properly [1,2]. International scientific societies have established recommendations regarding the compositions of nutritional mixtures [2,3,4,5]. Significantly, PN is also associated with the risk of excessive intravenous administration of certain TEs present in PN products as contaminants, which can affect their concentration in serum and urine [6,7].

To our knowledge, there are no studies on the concentrations of arsenic (As) and cobalt (Co) in children receiving LPN. Both elements play a role in human metabolism; however, according to the World Health Organization, Food and Agriculture Organization, and International Atomic Energy Agency (WHO/FAO/IAEA), only Co belongs to the essential elements. Co is necessary for the proper growth and development of an organism [8].

Arsenic, commonly known as a highly toxic element, also has a biological role in the human body, although its metabolic function is not well understood [9]. 

Groundwater and various foods, including fish, seafood, and rice, are major sources of As [10,11,12,13]. Inorganic As is toxic and carcinogenic, in contrast to the nontoxic organic form found, for example, in seafood [12,13]. Chronic exposure to inorganic As at a dose of 10 µg/kg/day or more leads to arsenicism, which is characterized by skin cornification and changes in pigmentation. Acute arsenic poisoning can result in encephalopathy, gastrointestinal symptoms, anemia, liver damage, and cardiovascular diseases [13,14].

The International Agency for Research on Cancer (IARC) has recognized arsenic as a Group 1 carcinogen and established a causal role in human bladder, lung, and skin cancers [15]. The toxicity of As is associated with its reactivity and the generation of free radicals and sulfur-containing compounds. This element induces oxidative stress in the kidneys and can lead to the dysfunction of renal tubular epithelial cells [16].

Due to numerous adverse effects, As is not a component of any commercially available PN preparation and is only present as a contaminant. However, it seems very important to monitor its concentrations in both serum and urine, especially in children who receive PN. It should be emphasized that in a study by Bohrer et al., which determined the As contents in PN preparations, all commercial preparations contained this element to some extent, but the total As contents only exceeded the permissible limit in a few samples [17]. In another study conducted on newborns briefly receiving parenteral nutrition, all samples of mixtures used in PN were contaminated with metals, with 96% exceeding the critical As limit (0.3 µg/kg body weight/day) [18].

Cobalt (Co) is found in solutions used for parenteral nutrition. Both its deficiency and excess can lead to various complications; therefore, monitoring its levels in both urine and serum seems to be extremely important. It is an essential element for forming vitamin B12 (hydroxocobalamin), which acts as a cofactor for enzymes in metabolic pathways crucial for mitochondrial function, DNA synthesis, neurotransmitter production, myelin sheath formation in neurons, and blood cell formation [2]. Over the past three decades, the anticancer potential of Co complexes has been studied extensively, and much effort has been devoted to understanding their mechanisms of action [19].

On the other hand, Co is also known for its carcinogenic properties. The last update by the IARC regarding Co and its compounds was published in 2006, classifying metallic cobalt with tungsten as probably carcinogenic to humans (Group 2A) [20]. Moreover, some of its compounds, such as cobalt sulfate and chloride, are classified in Group 2A by the European Union (EC Regulation 1272/2008). Some epidemiological studies have reported an increased risk of lung cancer among workers exposed to Co with tungsten carbide in the hard metal industry [21]. Other documented adverse health effects concern the respiratory tract and cardiovascular system, with Co-induced cardiomyopathy being particularly dangerous [20]. The main sources of Co in the population are diet and industrial exposure, with the average consumption ranging from 5 to 45 µg/day [2]. 

Currently, only one product provides Co in PN (Decan^®^, Laboratories Aguettant, Lyon, France), and it is not used in children. In addition, as supplementation with vitamin B12 is recommended for patients receiving PN [2,4], Co supplementation is unnecessary. Co is therefore present in PN products only as a contaminant [18,22]. In a study by Pluhator-Murton et al., Co was detected in PN preparations, and the storage time and temperature significantly influenced the concentrations of Co and some other trace elements [22].

Since we revealed interesting results regarding aluminum concentrations in children receiving PN, we decided to analyze additional elements (As and Co), which, like aluminum, constitute contamination in the mixtures used in PN. Knowledge about the status of As and Co in children receiving parenteral nutrition is scarce. Therefore, our study aimed to determine the impact of LPN in children on the serum levels of As and Co and the excretion of these elements in urine. Children receiving LPN were compared with a control group of healthy children. Results concerning the same group of patients regarding the aluminum concentrations delivered intravenously in PN mixtures were presented by us in 2023 [6]. 

## 2. Materials

### Study Participants

This study was a continuation of a previously published article. It was based on the same group of patients; however, it considered different elements. The previous study was based only on aluminum concentrations [6]. It involved patients from two centers in Poland conducting a home parenteral nutrition (HPN) program. In total, 83 minors (31 girls and 52 boys) from all over Poland, aged 7.3 months to 18 years, received LPN between 2004 and 2022. The duration of PN ranged from 4.6 months to 16.75 years. The reasons for implementing PN included short bowel syndrome (88% of cases), intestinal motility disorders (7% of cases), and enteropathy (5% of cases). 

All patients received nutritional mixtures with macronutrients, trace elements, and vitamins. The compositions of the mixtures were personalized for their individual needs based on their ages, body weights, underlying diseases, and overall conditions. 

In the study group, the serum levels of As and Co were examined in all 83 patients, and the excretion of these elements in urine was assessed in 74 children. The control group consisted of 121 participants (54 girls and 67 boys); the serum levels of As and Co were determined in 121 children, while the excretion of these elements in urine was assessed in 114 participants. The storage times for the serum and urine samples were similar in both groups, ranging from 6 to 22 months. Samples of serum and urine were collected from both the study and control groups in 2021 and 2022.

The study group and the control group were similar in terms of age and sex. Based on data on the time required for arsenic elimination [23], participants in the control group abstained from consuming fish and seafood for at least five days before sample collection. 

All study participants (control and study groups) underwent clinical examinations, and their body weights and heights were measured. The measurement values were compared to WHO centile charts (https://www.ptzkd.org/new/standardy-i-zalecenia/; URL accessed on 10 March 2024). The control group included children with normal anthropometric parameters. None of the study participants showed clinical symptoms of infection or toxic effects of As or Co. The supporting information can be found in Table S1 (https://docs.google.com/spreadsheets/d/1o88TmgRtqQrQRj6ZOKhiYgobO_dZwVai/edit?usp=share_link&ouid=118126989599175476191&rtpof=true&sd=true; accessed on 10 March 2024).

## 3. Methods

The current study was conducted according to the earlier methodology [6].

### 3.1. Test Tubes

To guarantee the highest level of correctness, several types of blood collection tubes were studied to control background element levels. BD Vacutainer #454001 tubes were carefully chosen for collecting urine samples and the blood samples used to formulate serum. 

### 3.2. Determination of As and Co

As described in the previous article [6], the blood samples used to formulate serum were obtained from fasting patients through venipuncture using a Vacutainer^®^ System (BD EST Z #362725, Plymouth, UK). Subsequently, the tubes were centrifuged at 1300× *g* for 12 min. Following centrifugation, the serum was carefully divided into new cryovials and then frozen at −80 °C until analysis. Similarly, the urine samples were aliquoted into new cryovials immediately after collection and stored at −80 °C until analysis. The urine samples were centrifuged at 5000× *g* for 5 min before analysis.

According to the methodology of the previous study [6], the elemental compositions of the samples were analyzed using ICP-MS with specific parameters. Calibration standards were prepared using a stock solution, and the metal levels in the urine were normalized using the metal-to-creatinine ratio.

### 3.3. Quality Control

As described in the previous article [6], the correctness and precision of the measurements were estimated using certified reference materials (CRMs): ClinChek^®^ Plasmonorm Serum Trace Elements Level 1 (Recipe, Munich, Germany) for the serum samples and ClinChek^®^ Urine Control Level 1 (Recipe, Munich, Germany) for the urine samples. The technical particulars, plasma operating backgrounds, and mass spectrometer acquisition parameters can be provided upon request. 

### 3.4. Statistical Analysis

The Shapiro–Wilk test was used to assess the normality of the data distribution and indicated that the data did not follow a normal distribution. Descriptive statistics, such as the medians and interquartile ranges, were employed to summarize the analysis results. A non-parametric Mann–Whitney U test (U M-W) was applied for data analysis. The correlations between the variables were evaluated using the Spearman rank-order correlation test. A significance level of *p* < 0.05 was adopted to determine statistical significance. 

## 4. Results

**Figure 1:**
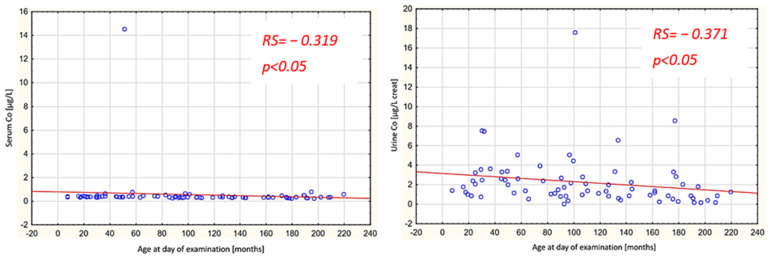
Serum and urine Co levels with respect to age in the study group. RS: Spearman’s rank correlation coefficient; blue circles: separate values for each patient, red line: line of regression.

**Figure 2:**
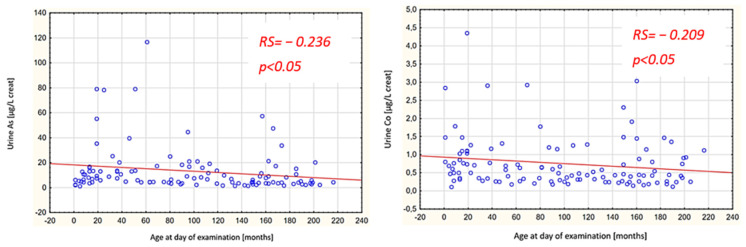
Urine Co and As levels with respect to age in the control group. RS: Spearman’s rank correlation coefficient; blue circles: separate values for each patient, red line: line of regression.

The research findings are summarized in Table 1 and Table 2 and Figure 1 and Figure 2.

### 4.1. Serum and Urine As and Co Levels in the Study and Control Groups

Table 1 presents the serum and urine levels of As and Co in the study and control groups. The serum levels are expressed in µg/L, while the urine levels are expressed in µg/g creatinine.

No statistically significant difference between the study and control groups was observed for the serum As levels. However, the serum Co concentration was statistically higher in the study group (medians of 0.37 vs. 0.29, *p* < 0.001). Comparing the As and Co concentrations in the urine between the study and control groups, statistically significant differences were observed in each case (medians of 4.59 vs. 6.97 and 1.46 vs. 0.5, respectively, for As and Co in the study and control groups) (Table 1). The results indicated increased Co values in the urine of patients receiving parenteral nutrition compared to the control group, while for As, the results were opposite.

### 4.2. Sex-Dependent Differences in Serum and Urine As and Co

Table 2 compares the As and Co levels in the serum and urine depending on sex in both the study and control groups.

The sex-dependent differences in the As and Co levels in the serum and urine were analyzed in both the study and control groups. The male patients receiving LPN had higher serum As concentrations and urinary excretion than the females. No statistically significant sex-related differences regarding the serum and urine Co levels were found in the study group. The control group showed no significant sex-dependent differences in the As and Co levels in the serum and urine.

### 4.3. Age-Dependent Differences in Serum and Urine As and Co 

Spearman rank-order correlations were examined concerning the relationships between the levels of the investigated elements and age. In the study group, statistically significant negative correlations were noted between age and the concentration of Co in both the serum and urine, while in the control group, a statistically significant negative correlation between age and the concentration of Co was only observed in the urine. Additionally, a statistically significant negative correlation between age and the concentration of As in the urine was observed in the control group (Figure 1 and Figure 2).

## 5. Discussion

Since we obtained unexpected results regarding aluminum concentrations in blood and urine in children receiving PN [6], we decided to perform further research based on analysis of As and Co. In the previous data, we revealed that healthy children had higher serum aluminum concentrations than those in the parenteral nutrition group, which may indicate the influence of one’s environment and diet on aluminum serum levels. To our knowledge, there are no publications on the levels of As and Co in children receiving LPN.

### 5.1. Arsenic

The number of studies assessing the toxicity of As in children exposed to environmental factors is considerably higher than the number assessing this topic in patients receiving PN [24]. A study by Alao et al., evaluating the As excretion in the urine of 465 children in Bangladesh exposed to this element in contaminated drinking water, showed an increased risk of stunting and weight loss in children with urinary As levels of 31 μg/L and above [10]. Gardner et al. showed lower weight gain in children exposed to As [25], while Rahman et al. conducted an extensive review of studies assessing human exposure to increased As levels in groundwater and its associated complications [11]. 

In our study, the concentration of As in the serum of patients receiving LPN was lower (median of 0.63 µg/L, range of 0.51–3.04 µg/L) than in the control group (median of 0.65 µg/L, range of 0.37–8.91 µg/L); however, this difference was not statistically significant (Table 1). These results are similar to the serum As levels determined in a study conducted in Poland in a group of 31 healthy children aged 2–16 years, which amounted to 0.25 ± 0.35 µg/L [26]. 

We could not find any reports assessing serum As levels in large groups of children. The norms for persons aged 6–19 years published by the Canadian Health Measures Survey concern As levels in whole blood [18], similar to a study that determined the As levels in the Flemish population [12]. In a study conducted in Brazil that determined the reference levels of As in healthy adults (cited by us due to the large study group and the determination of As in serum), the mean levels of As in serum were 1.153 µg/L in men and 1.195 µg/L in women. Similar to our control group, there was no statistically significant difference between women and men [27]. In our study group, however, we found that male patients had higher levels of As in serum and higher excretion in urine than female patients (Table 2). In the control group, we found a concurrent age-dependent decrease in the urinary As concentration (Figure 2).

Concerning the article cited above, the results obtained from our patients and the healthy children in the control group may seem optimistic. They correspond to the results obtained in a study by Al-Saleh, where no increased As levels were found in the serum of preterm infants receiving PN [18]. In Jacobson and West’s 1977 study assessing trace element balances in four adult patients receiving short-term PN, all showed negative As balances [28].

The excretion of As in urine in our study was significantly higher in the control group compared to the study group (the median levels were 6.97 µg/g creatinine and 4.59 µg/g creatinine, respectively) (Table 1). For comparison, in the Flemish population study, the geometric mean of As in the urine of young people aged 14–15 was 9.3 µg/g creatinine; in adults, it was 15.9 µg/g creatinine and was higher in women [12]. In the large German Environmental Survey (GerES III) study, As excretion in urine was determined to be 3.08 µg/g creatinine in adults [29]. In the U.S. National Health and Nutrition Examination Survey (NHANES) study of the population aged 6–19, urinary As levels ranged from 6.06 to 8.97 µg/g creatinine [30]. The reference values for As in urine in healthy adults in the Brazilian study were as follows: the mean in women was 11.34 µg/g creatinine and the mean in men was 12.48 µg/g creatinine. Similar to our control group, there was no statistically significant difference between women and men (Table 2) [27]. 

The levels of As in the urine of all our studied children were much lower than in most of the cited studies, except for the German study, and fell within the range provided by the NHANES. Significantly higher excretion of this element in the control group may indicate lower exposure to As in patients receiving PN. It can be inferred that contaminants present in PN mixtures exposed patients to As to a lesser extent than environmental factors affecting the control group of healthy children receiving regular diets.

### 5.2. Cobalt

The literature on Co levels in children includes studies on this element’s status in children with iron deficiency anemia [31], severe malnutrition [32], and hypertension [33]. In the available literature, we found one study, which was already mentioned, on the serum and urine Co balance in four adult patients receiving PN. It showed slight Co retention among other trace elements, and the serum Co concentrations decreased in three of the patients during PN compared to the baseline values [28]. There is a lack of studies that assess Co levels in children receiving PN.

In our study, patients receiving LPN showed a significant increase in the Co concentration in serum and increased excretion of this element in urine compared to the control group (medians of 0.37 µg/L vs. 0.29 µg/L (*p* < 0.001) and 1.46 µg/g creatinine vs. 0.5 µg/g creatinine (*p* < 0.001), respectively, in serum and urine in the study and control groups). Greater urinary Co excretion reflected higher serum concentrations in the study group. There were no significant differences between men and women in the study and control groups (Table 2).

For comparison, in a study conducted in China that determined trace element levels in serum in over 2200 healthy children, the reference range for Co was between 0.07 and 0.73 µg/L [34]. In a Brazilian study involving 240 adults, the mean serum Co concentrations were 0.15 µg/L in men and 0.158 µg/L in women [27]. In both cited studies, similar to our control group (Table 2), no statistical differences in serum Co were found between men and women. 

Unfortunately, there is a lack of data for determining reference ranges for serum Co levels in healthy children from European countries. The higher Co levels in the serum of children receiving LPN in our study indicate a sufficient supply of this element from contaminants in PN products, demonstrating that additional substitution is unnecessary. At the same time, concerning the cited Chinese study, these levels do not exceed the reference range, so there seems to be no risk of Co toxicity. Considering the role of vitamin B12 as a cofactor in many metabolic pathways, the metabolism of which depends to a large extent on Co, substitution of this vitamin is recommended for patients receiving LPN [4].

The median Co concentration in urine in the study group was higher than that recorded in 4-year-old healthy children from Austria in a study by Grimalt et al. (1.0 µg/g creatinine) [35], similar to that observed in a study of children aged 6–11 from Spain (1.4 µg/g creatinine) [36], and higher than the U.S. norms for the population aged 3 to 19 years (0.465–0.974 µg/g creatinine) [30]. 

In the control group, the urine Co levels were within the norms given in the NHANES report. It should be noted that in the report mentioned above, urine Co levels decrease with a child’s age [30]. The same relationship was shown in our study in both the study and control groups (Figure 1 and Figure 2). Moreover, in the study group, the serum Co level also decreased with age (Figure 1). This result corresponds to the cited Brazilian study in which serum Co levels in adults were significantly lower than the reference ranges for children published based on the Chinese study [27,34]. This age-dependent decrease in Co levels can be explained by a shift in the distribution of this element in the body, which is primarily associated with bone metabolism. A study by Chang et al. showed that the Co concentration in bones decreases with age. This is caused by the migration of Co from the inner bone to the outer bone, which prevents osteopenia [37]. 

While the age of the population in the literature data that we cite does not perfectly align with our study group’s age, the research we cite nonetheless provides a broader context for interpreting our results. We acknowledge that populations in different regions exhibit different environmental exposure profiles, and research results should be referenced within their respective reference ranges. However, since specific reference ranges are unavailable for elements such as As and Co in children in Poland, we referred to the results in the control group.

## 6. Conclusions

Children receiving LPN are not exposed to As, and even though the concentration of Co in serum and urine was higher in the LPN group than in healthy controls, neither trace element poses a health threat to children requiring LPN.

## Figures and Tables

**Table 1:** Serum and urine As and Co levels in the study and control groups.

Trace Element		Group Size	Median	Range [Min–Max]	Lower Q	Upper Q	*p*-Value
As in serum [µg/L]	Study group	83	0.63	0.51–3.04	0.59	0.77	NS
	Control group	121	0.65	0.37–8.91	0.58	0.78	
Co in serum [µg/L]	Study group	83	0.37	0.24–14.54	0.33	0.42	*p* < 0.001
	Control group	121	0.29	0.16–0.79	0.24	0.34	
As in urine [µg/g creatinine]	Study group	74	4.59	0.1–124.56	2.84	11.03	*p* < 0.01
	Control group	114	6.97	1.14–116.7	4.04	13.67	
Co in urine [µg/g creatinine]	Study group	74	1.46	0.03–17.6	0.88	2.72	*p* < 0.001
	Control group	114	0.5	0.11–4.35	0.3	1.03	

NS—statistically nonsignificant (*p* > 0.05).

**Table 2:** Serum and urine levels of As and Co in the study and control groups by sex.

Trace Element	Gender	Group Size	Median	Range [Min–Max]	Lower Q	Upper Q	*p*-Value
**Study group**							
As in serum [µg/L]	Female	31	0.61	0.51–2.32	0.57	0.72	*p* < 0.05
	Male	52	0.63	0.55–3.04	0.6	0.8	
As in urine [µg/g creatinine]	Female	28	3.5	0.87–21.85	2.16	6.28	*p* < 0.05
	Male	46	5.73	0.1–124.55	3.15	14.24	
Co in serum [µg/L]	Female	31	0.37	0.27–0.77	0.34	0.42	NS
	Male	52	0.36	0.24–14.54	0.32	0.43	
Co in urine [µg/g creatinine]	Female	28	1.37	0.2–7.53	0.94	2.44	NS
	Male	46	1.81	0.03–17.6	0.87	3.31	
**Control group**							
As in serum [µg/L]	Female	54	0.66	0.51–1.9	0.6	0.77	NS
	Male	67	0.61	0.37–8.9	0.57	0.85	
As in urine [µg/g creatinine]	Female	51	6.89	1.36–79.23	4.28	16.07	NS
	Male	63	7.05	1.15–116.71	3.68	13.10	
Co in serum [µg/L]	Female	54	0.28	0.16–0.79	0.25	0.38	NS
	Male	67	0.3	0.16–0.54	0.24	0.33	
Co in urine [µg/g creatinine]	Female	51	0.58	0.18–4.35	0.32	1.16	NS
	Male	63	0.48	0.11–3.03	0.27	0.89	

NS—statistically nonsignificant (*p* > 0.05).

## Data Availability

The data presented in this study are available upon request from H.R.

## Supplementary Materials

The following supporting information can be downloaded at https://docs.google.com/spreadsheets/d/1o88TmgRtqQrQRj6ZOKhiYgobO_dZwVai/edit?usp=share_link&ouid=118126989599175476191&rtpof=true&sd=true, Table S1: Database of As and Co—study group and control group.
